# A galactoarabinan-containing glycoprotein isolated from the fruits of *Lycium barbarum* reverses the tumor-associated macrophage phenotype

**DOI:** 10.3389/fphar.2025.1593407

**Published:** 2025-10-29

**Authors:** Bo Wang, Bowen Lu, Junqiang Lv, Caixia Dong, Changcai Bai

**Affiliations:** ^1^ Department of Pharmacy, People’s Hospital of Ningxia Hui Autonomous Region, Ningxia Medical University, Yinchuan, China; ^2^ College of Pharmacy, Ningxia Medical University, Yinchuan, China; ^3^ Key Laboratory of Ningxia Ethnomedicine Modernization, Ministry of Education, Ningxia Medical University, Yinchuan, China; ^4^ Department of Pharmacognosy, College of Pharmacy, Jiamusi University, Jiamusi, China; ^5^ Tianjin Key Laboratory of Cellular and Molecular Immunology, Key Laboratory of Educational Ministry of China, School of Basic Medical Sciences, Tianjin Medical University, Tianjin, China; ^6^ Tianjin Key Laboratory of Technologies Enabling Development of Clinical Therapeutics and Diagnosis, School of Pharmacy, Tianjin Medical University, Tianjin, China

**Keywords:** *Lycium barbarum*, polysaccharide, tumor-associated macrophage, immunomodulatory activity, colorectal cancer

## Abstract

*Lycium barbarum* fruits possess a well-established history of use in traditional Chinese medicine due to their potent immunomodulatory properties, extending to cancer immunity. In this study, a purified galactoarabinan-containing glycoprotein, designated LBNP-1, with an apparent molecular weight of 56.2 kDa, was isolated from the aqueous extract of *L. barbarum* fruits. Our findings revealed that LBNP-1 possesses a branched arabinan structure as its saccharide moiety. Bioactivity assays demonstrated that LBNP-1 polarized M2 macrophages towards the M1 phenotype. Furthermore, the LBNP-1-induced macrophage phenotypic shift was inhibited by a TLR4 antagonist. Western blot analysis indicated that LBNP-1 activated the NF-*κ*B signaling pathway, thereby promoting polarization of tumor-associated macrophages (TAMs) towards the M1 phenotype. Moreover, conditioned medium derived from RAW264.7 cells pretreated with LBNP-1 exhibited anticancer effects against colorectal cancer (CRC) cells. Our research highlights the antitumor effect of LBNP-1 against CRC, mediated by reversing the TAM phenotype. LBNP-1 may represent a potential immunomodulator for the treatment of malignancies.

## 1 Introduction

Colorectal cancer (CRC) ranks as the third leading cause of global cancer-related mortality, with projections indicating approximately 153,020 new diagnoses and 52,550 deaths in 2023 (Siegel et al., 2023). It currently exhibits a substantial burden characterized by high incidence, recurrence, and mortality rates. Primary treatment modalities include surgery, combination chemotherapy, targeted therapy (including anti-EGFR agents), and antiangiogenic therapy. Although these therapies for CRC are established in research and clinical practice, many patients present with advanced-stage disease, precluding them from receiving curative-intent treatment. Unfortunately, a significant proportion of CRC patients experience recurrence despite undergoing radical surgery and standard adjuvant radiotherapy (Li Y. et al., 2022; Osterman and Glimelius, 2018). Conventional chemotherapeutic drugs also exhibit significant drawbacks, including poor solubility, inadequate permeability, non-specific targeting, limited tumor accumulation, and dose-dependent toxicity to healthy tissues (Din et al., 2017). This has spurred significant worldwide research into natural antitumor agents possessing low or negligible cytotoxicity as novel therapeutic approaches. The recent advent of strategies designed to activate or enhance the innate immune system for cancer cell recognition and elimination represents a revolutionary shift in cancer therapy. Therefore, natural antitumor agents possessing immunomodulatory properties have become a critical area of investigation.

As key components of innate immunity, macrophages are vital for cancer control. These cells represent a heterogeneous population existing in multiple functional states throughout tissues. Functionally, macrophages polarize into two distinct subsets: classically activated M1 macrophages and alternatively activated M2 macrophages. Polarization by LPS or Th1 cytokines (such as IFN-γ) induces M1 macrophages to produce proinflammatory cytokines, whereas Th2 cytokines (including IL-4 and IL-13) drive M2 macrophage polarization and the secretion of anti-inflammatory cytokines (Pan et al., 2020). M1/M2 macrophage polarization plays a critical role in tumor progression, with its balance subject to modification by tumor microenvironment (TME) dynamics or therapeutic strategies. Tumor-associated macrophages (TAMs), which exhibit an M2-like phenotype, represent a significant component of the immune cell infiltrate in the TME. TAMs drive pro-tumor functions such as: angiogenesis and lymphangiogenesis; immunosuppression; hypoxia-induced proliferation; metastasis; and therapeutic resistance. In contrast, M1 macrophages display potent anti-tumor activity. Compelling evidence indicates that TME-mediated immunosuppression significantly impedes the clinical efficacy of immunotherapies (Xiang et al., 2021). Therefore, targeting TAMs or modulating M2 macrophage polarization in the TME constitutes a promising avenue for advancing tumor immunotherapy.

Mounting evidence indicates natural polysaccharides function as effective immunomodulators, demonstrating immunostimulatory activity alongside favorable safety profiles characterized by low toxicity and minimal side effects (Pu et al., 2022; Yuan et al., 2022). *Lycium barbarum* fruit, commonly termed goji berry or wolfberry, represents a prominent traditional Chinese medicinal (TCM) substance and esteemed tonic. In TCM practice, it addresses manifestations of liver and kidney deficiency in aging populations, such as dizziness, fatigue, anorexia, and sleep disturbances. Furthermore, it is reputed to tonify the liver and kidneys, nourish essence, and improve eyesight.

Accumulated research demonstrates immunomodulatory properties (Wang et al., 2021), anticancer effects (Sun et al., 2022) and hypoglycemic activities (Tang et al., 2015; Zhou et al., 2022) in *L. barbarum* polysaccharides. Several polysaccharides alter macrophage polarization states and display antitumor effects. Notably, *Lepidium meyenii*-derived maca polysaccharide induces M2-to-M1 macrophage repolarization (Zhang et al., 2017). Galactan from *Cantharellus cibarius* drives M1-like polarization in tumor-associated macrophages, inhibiting cancer cell proliferation via TLR2 activation (Meng et al., 2019). The potential of *L. barbarum* polysaccharides to boost antitumor immunity via M2-like to M1-like macrophage transformation remains unexplored.

We extracted a galactoarabinan-containing glycoprotein (LBNP-1) from *L. barbarum* and determined its structural features through fourier transform infrared spectroscopy (FT-IR), methylation, gas chromatography‒mass spectrometry (GC-MS), and nuclear magnetic resonance spectroscopy (NMR). We evaluated LBNP-1’s immunomodulatory function by measuring M2-to-M1 phenotypic conversion in RAW264.7 macrophages. Our findings show LBNP-1 suppresses tumor cell proliferation by reversing TAM immunosuppression through M1 repolarization. This polarization effect likely represents the underlying antitumor mechanism. Our work provides mechanistic insights into *L. barbarum* polysaccharide immunomodulation of TAMs, supporting broader therapeutic utilization.

## 2 Materials and methods

### 2.1 Reagents

Dried goji berries were obtained from Ningxia Golden Sun Pharmaceutical Co., Ltd. (Yinchuan, China; Batch No. 20171020) and were authenticated by Professor Changcai Bai from Ningxia Medical University College of Pharmacy (Yinchuan, China) as the fruit of *L. barbarum*. The samples were stored in the Engineering Technology Center of Ningxia Medical University. Recombinant murine interleukin 4 (IL-4) was purchased from PeproTech (United States), and LPS was obtained from Sigma‒Aldrich (St. Louis, MO). The TLR4 inhibitor TAK-242 and the TLR2 inhibitor C29 were obtained from Selleck Chemicals (Houston, United States). A cell counting kit-8 (CCK-8) was purchased from New Saimei Biotechnology Co., Ltd. (Suzhou, China). All the other chemicals used were of analytical grade.

### 2.2 Extraction, isolation and purification of polysaccharides

**FIGURE 1:**
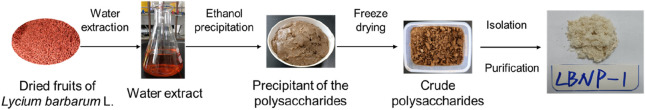
Schematic diagram of the extraction, separation and purification of polysaccharides from *Lycium barbarum* L.

Extraction, isolation and purification of LBNP-1 was performed using the previous method (Sun et al., 2019) with simple modifications. The procedure for extracting and purifying polysaccharides from Ningxia *L. barbarum* is illustrated in Figure 1. Dried goji berries (5.0 kg) were ground into a powder for polysaccharide extraction. The powder was then mixed with water at a ratio of 10:1 (mL/g) and subjected to three extractions at 60 °C for 2 h each. The combined aqueous extracts were filtered, and the resulting filtrate was concentrated under vacuum to approximately 1/4 of the original volume. Ethanol (95%) was added to precipitate the polysaccharides, and the mixture was left overnight at 4 °C. After centrifugation, the supernatant was collected, and the precipitate was redissolved in distilled water. The dissolved solution was then subjected to centrifugation, dialysis (7,000 Da cutoff), and freeze-drying to obtain the crude polysaccharide fraction known as *L. barbarum* crude polysaccharide (LBCP).

LBCP (1.5 g) was loaded onto a DEAE-cellulose column and eluted with deionized water. The 20 mL eluate was collected in a separate tube, and the carbohydrate content was analyzed via UV absorption at 280 nm and via a phenol sulfuric acid assay at 490 nm. The obtained fraction was denoted as LBN and was accurately weighed and diluted with a 0.1 M sodium chloride mobile phase to a final volume of 20 mL. The mixture was then centrifuged, and the supernatant was collected. Next, the supernatant was purified using a gel column (2.6 × 100 cm) packed with sepharose-6B (GE). The mobile phase consisted of 0.1 M sodium chloride, and the flow rate was set at 1.0 mL/min to obtain three fractions: LBN-1, LBN-2, and LBN-3. The collected liquid was concentrated using a rotary evaporator and freeze-dried to obtain the most abundant component, LBN-2. LBN-2 was further purified using a Sephacryl S-300 HR column (2.2 i.d. × 100 cm) to obtain the purified polysaccharide LBNP-1 (323.7 mg). Each fraction (5 mL) was collected and analyzed using the phenol-H_2_SO_4_ method at 480 nm and UV absorbance spectroscopy at 280 nm.

### 2.3 Colorimetric analysis

The total carbohydrate content was determined using the phenol-H_2_SO_4_ method (DuBois et al., 1956). Galactose was used as the standard for comparison. The protein content was measured using a Bio-Rad protein assay kit with BSA serving as the standard protein.

### 2.4 Homogeneity and molecular weight analysis

The homogeneity and molecular weight analysis of LBNP-1 was studied with reference to the method of Zhang et al. (2019). The homogeneity of the sample and its apparent molecular weight were determined using HPGPC with an Agilent 1260 instrument. The column temperature was set at 40 °C, and the samples (2 mg/mL) were loaded onto a PL Aquagel-OHMIXED-H column (7.5 mm × 300 mm). Elution was performed with 0.02 M ammonium acetate at a flow rate of 0.6 mL/min. To construct the calibration curve, commercially available standard molecular markers (Pullulan P-5, P-10, P-20, P-50, P-100, P-200, P-400, and P-800) were used. The apparent molecular weights were calculated by utilizing the calibration curve derived from the pullulan standards.

### 2.5 Monosaccharide composition analysis

Monosaccharides were analyzed through HPLC following strong acidolysis and derivatization with 1-phenyl-3-methyl-5-pyrazolone (Susumu Honda et al., 1989). A 2 M trifluoroacetic acid (TFA) solution was utilized to hydrolyze the sample (1 mg) for 2 h at 120 °C. After depressurization using a rotary evaporator to remove TFA, the hydrolysate was dissolved in water. Next, the residue was treated with an aqueous solution of 0.6 M NaOH and 0.5 M PMP-CH_3_OH, and the reaction was conducted at 70 °C for 30 min. The reaction product was neutralized with 0.3 M HCl, and any excess PMP was eliminated using chloroform. The resulting aqueous phase was filtered through a 0.22 μm nylon membrane and then loaded onto an XDB-C18 column (4.6 mm × 250 mm, 5 μm; Agilent). With a flow rate of 0.17 mL/min, a mobile phase consisting of acetonitrile and 0.1 M phosphate buffer was used. The monosaccharide sequences identified in LBNP-1 were compared to the retention times of standard sugars.

### 2.6 Amino acid composition analysis

Amino acid composition analysis was carried out according to reported literature with some modifications (Qin et al., 2021). The sample (1 mg) was completely dissolved in 1 mL of 6 mol/L HCl and heated at 110 °C for 22 h. After cooling, nitrogen was added to remove the HCl at 50 °C, and the sample was then redissolved in 1 mL of 0.02 mol/L HCl. The analysis was carried out using an Automatic Amino Acid Analyzer (Hitachi, Japan).

### 2.7 Fourier transform infrared (FT-IR) spectral analysis

Glycosidic bond of LBNP-1 was determined by FT-IR spectrometer (Nicolet Nagna-IR 550) method according to the literature (Liu et al., 2022). To prepare the samples for FT-IR analysis, 200 mg of potassium bromide was mixed with the sample (2 mg) and crushed into tablets. The control sample consisted of powdered potassium bromide. An FT-IR spectrometer (Nicolet Nagna-IR 550) operating in the vibration range of 4,000–400 cm^−1^ was utilized for the analysis. A resolution ratio of 32 scans was employed.

### 2.8 Methylation and GC‒MS analysis

The methylation and GC‒MS experiment were carried out according to the literature (Li et al., 2021). Methylation analysis of the sample was conducted using the method described in the literature (Ciucanu and Kerek, 1984). The sample (2 mg) was dissolved in 1 mL of DMSO, and then powdered NaOH (40 mg) was added and blended at room temperature. Iodomethane was added to the mixture three times, with 15 min between each addition. The reaction was stopped by adding distilled water, followed by chloroform extraction. After posttreatment, the resulting compounds were reduced with NaBD_4_ and NH_3_-H_2_O (1 mol/L) at room temperature for 24 h and then hydrolyzed with TFA (2 mol/L) at 121 °C for 90 min. Alditol acetates that had been partially methylated were produced through acetylation with acetic anhydride. The GC‒MS apparatus used for the synthesis and analysis of the partly methylated alditol acetates was equipped with a quadrupole detector and an HP‒5 MS fused quartz capillary column. The GC oven temperature program was set to an initial temperature of 120 °C and then increased to 280 °C at a rate of 4 °C/min for 5 min using helium as the carrier gas. The molar ratio of each residue was calculated and expressed as the percentage peak area.

### 2.9 NMR spectroscopy analysis

The structure of LBNP-1 were detected using the NMR spectrometer (Sun et al., 2019). A 100 mg sample was dissolved in 0.55 mL D_2_O. The sample was analyzed using a high-resolution NMR spectrometer (AVANCE III HD 400, Bruker, Germany) at room temperature. The structural characteristics of the sample were determined on a Varian INOV A 300 NMR spectrometer.

### 2.10 Cell culture

The protocol for RAW 264.7 macrophage cell culture followed a standard laboratory method (Liu et al., 2021). Briefly, RAW 264.7 cells (Tianjin Medical University, Tianjin, China) were cultured in DMEM supplemented with 10% FBS, 100 U/mL penicillin, and 100 μg/mL streptomycin at 37 °C in a CO-150 incubator (New Brunswick Scientific) with a CO_2_ atmosphere of 5%.

### 2.11 Induction of macrophage polarization

Induction of macrophage polarization was carried out according to a previously described method by Ren et al. (2019) with minor modeifications. RAW 264.7 cells were plated in 6-well plates at a density of 8 × 10^5^ cells/well according to the experimental purpose. M1 or M2 macrophages were generated by treating RAW264.7 cells with 1.0 μg/mL LPS or 20 ng/mL IL-4 for 12 h. Then, the treated RAW264.7 cells were incubated with LBNP-1 (100 μg/mL) for 24 h. In the inhibitor experiment, M2 macrophages were pretreated with C29 (20 μM) or TAK-242 (20 μM) for 2 h and then treated with LBNP-1 (100 μg/mL) for 24 h.

### 2.12 Conditioned medium preparation

The method of conditioned medium preparation was according to previous literature (Zong et al., 2020). RAW264.7 cells were treated with 20 ng/mL IL-4, with or without various concentrations of LBNP-1, for 12 h to obtain polarized macrophages. Cell culture supernatants were collected and filtered through a 0.45 μm filter (Merck Millipore, Billerica, MA, United States) to obtain conditioned medium (CM).

### 2.13 Cell viability assay

The viability of RAW264.7 cells treated with different concentrations of LBNP-1 was determined using the CCK8 assay (Pu et al., 2022). In brief, the cells were cultured in a 96-well plate at a density of 4 × 10^4^ cells per well. After treatment with LBNP-1 for 24 h, 10 μL of CCK-8 reagent was added to each well. The cells were then incubated for an additional 2 h at 37 °C. The absorbance was measured at 450 nm using a microplate reader.

### 2.14 qRT‒PCR analysis

Total RNA was isolated from cultured cells using TRIzol reagent (TIANGEN, Beijing, China) following the manufacturer’s instructions. For qRT‒PCR analysis, 1 μg of RNA sample was reverse transcribed into cDNA using a reverse transcription kit (TaKaRa, Beijing, China). The primers used were synthesized by Sangon Biotech (Shanghai, China). The reaction mixtures containing SYBR Green (Roche, Basel, Switzerland) were prepared according to the manufacturer’s protocol. The expression levels of the target genes were normalized to those of the control gene Actin. The sequences of primers used for qRT‒PCR were as follows:

**Table:** 

Gene name	Primer	Sequence
mIL1-*β*	Forward	GCC​ACC​TTT​TGA​CAG​TGA​TGA​G
	Reverse	AAG​GTC​CAC​GGG​AAA​GAC​AC
mIL-6	Forward	GAC​AAA​GCC​AGA​GTC​CTT​CAG​A
	Reverse	GAG​CAT​TGG​AAA​TTG​GGG​TAG​G
mTNF-*α*	Forward	GCA​CAG​AAA​GCA​TGA​TCC​GC
	Reverse	GTT​TGC​TAC​GAC​GTG​GGC​T
mArg-1	Forward	TCG​GAG​CGC​CTT​TCT​CAA​AA
	Reverse	CAC​AGA​CCG​TGG​GTT​CTT​CA
mACT	Forward	ATG​TGG​ATC​AGC​AAG​CAG​GA
	Reverse	GGG​TGT​AAA​ACG​CAG​CTC​AG
mCD206	Forward	TTG​CCG​TCT​GAA​CTG​AGA​TGG
	Reverse	CAT​GAG​GCT​TCT​CCT​GCT​TCT
miNOS	Forward	CGG​CAA​ACA​TGA​CTT​CAG​GC
	Reverse	GCA​CAT​CAA​AGC​GGC​CAT​AG

### 2.15 Flow cytometry assay

M1 macrophages were characterized using CD86 (BioLegend, United States of America). The cell suspensions were stained with anti-CD86-FITC for 30 min in the dark at room temperature. The cells were then washed twice with PBS and resuspended in PBS. Finally, single-cell suspensions were acquired and analyzed using flow cytometry.

### 2.16 MC-38 cells and RAW264.7 cell coculture

The method of MC-38 cells and RAW264.7 cell coculture were according to previous literature (Jiang et al., 2024). RAW264.7 cells were treated with IL-4 (20 ng/mL) and LPS (1.0 μg/mL) and induced to differentiate into M1 or M2 macrophages. The M2 cells were then treated with LBNP-1 (100 μg/mL) for 12 h. The supernatant was obtained and filtered through a 0.22 μm filter (Merck Millipore, Billerica, MA, United States). MC-38 cells were seeded in a 96-well plate for 24 h and then cocultured with the supernatant.

### 2.17 Western blot

Western blotting was performed according to a previously described standard protocol (Guo et al., 2022). RAW264.7 cells in the logarithmic growth phase were seeded in a 6-well plate at a density of 2 × 10^6^ cells per well and cultured for 24 h. The cells were then polarized into M2-type macrophages using IL-4 (20 ng/mL) and treated with LBNP-1 for 15, 30, or 60 min. After discarding the culture medium, the cells were washed three times with cold PBS and lysed with RIPA lysis buffer. The protein concentration was determined using a BCA protein assay kit (Thermo Fisher Scientific). A total of 25 μg of protein from each sample was separated via sodium dodecyl sulfate‒polyacrylamide gel electrophoresis (SDS‒PAGE) and transferred to a polyvinylidene fluoride (PVDF) membrane. The membrane was blocked with 10% skim milk powder at room temperature for 2 h and then washed with TBST five times. The cells were then incubated with rabbit polyclonal antibodies (p-P65, P65, p-IκBα, and β-actin) at 4 °C overnight, followed by incubation with the anti-rabbit IgG secondary antibody at room temperature for 2 h.

### 2.18 Statistical analysis

The data are presented as the average ± SD from at least three independent experiments. Statistical analysis was performed using GraphPad Prism 8 software. One-way ANOVA was applied to statistical comparisons between groups. Student’s t-test was used to compare groups, with *P* values < 0.05 or 0.01 indicating statistical significance.

## 3 Results

### 3.1 Preparation of LBNP-1

The crude carbohydrate LBCP was isolated from *L. barbarum* through hot water extraction and 80% ethanol precipitation. The yield obtained was 3.4%. Subsequent ion-exchange chromatography on a DEAE 650 M column and gel permeation on Sepharose 6B and Sephacryl S-300 HR columns resulted in the purification of the main polysaccharide (LBNP-1) as a light-yellow‒white powder, with a mass of 323.7 mg.

### 3.2 Structural characterization of LBNP-1

**FIGURE 2:**
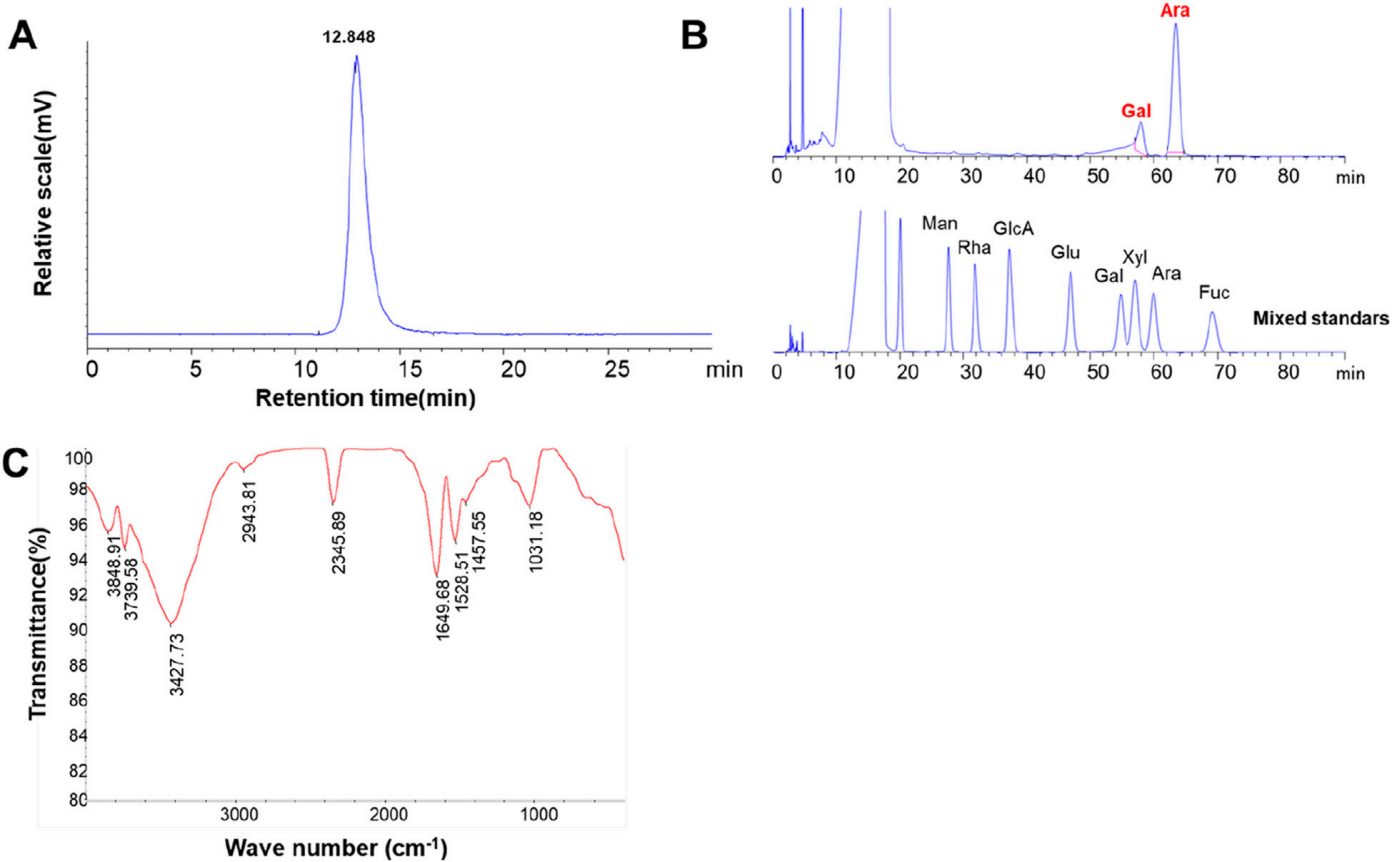
Preliminary structural estimate of LBNP-1. **(A)** HPGPC chromatograms of LBNP-1; **(B)** PMP-HPLC chromatogram analysis of the monosaccharide composition of LBNP-1; **(C)** FT-IR spectrum of LBNP-1; **(C)** GC‒MS analysis of LBNP-1 methylation. (Man, mannose; Rha, rhamnose; GlcA, glucuronic acid; GalA, galacturonic acid; Glc, glucose; Gal, galactose; Xyl, xylose; Ara, arabinose; Fuc, fucose).

The HPGPC profile of LBNP-1 is depicted in Figure 2A. A sharp and symmetrical peak was observed, indicating that the sample LBNP-1 was homogeneous based on its molecular weight distribution. The apparent molecular weight of LBNP-1 was determined to be 56.2 kDa. Table 1 presents the contents of total carbohydrates, uronic acids, and proteins. Results showed that LBNP-1 contain 36.4% of protein in addition to 29.2% of carbohydrates, indicating that LBNP-1 is a glycoprotein. Moreover, no uronic acid was contained in this sample.

**TABLE 1:** Chemical (%, w/w) and monosaccharide compositions (%, mol) of LBNP-1.

Polysaccharides	LBNP-1
Molecular weight (kD)	56.2
Chemical composition	
Total sugar (%)^a^	29.2
Uronic acid (%)^b^	-
Protein (%)^c^	36.4
Sugar composition	
Ara	81.2
Gal	18.8

^a^ Determined by phenol-sulfuric acid method.

^b^ M-hydroxybiphenyl method.

^c^ Bio-Rad protein assay kit.

### 3.3 Monosaccharide and amino acid composition of LBNP-1

The monosaccharide composition of LBNP-1 was analyzed via PMP-HPLC (Figure 2B). As shown in Table 1, LBNP-1 was found to be composed of Ara and Gal at a molar ratio of 4.3:1.0. Furthermore, the amino acid composition of LBNP-1 was determined, and the results are summarized in Table 2. Aspartic acid, serine, glutamic acid, proline, isoleucine, and tyrosine were the major amino acids in LBNP-1, accounting for 3.71%, 7.43%, 4.12%, 6.72%, 3.54%, and 4.23%, respectively. During the hydrolysis process, asparagine and glutamine are converted to aspartic acid and glutamic acid, respectively.

**TABLE 2:** Amino acid composition of the purified LBNP-1 from *L. barbarum* L.

Amino acid composition^a^	LBNP-1 (mg/g)
Asp	3.7
Thr	2.2
Ser	7.4
Glu	4.1
Pro	6.7
Gly	2.8
Ala	2.6
Cys	0.0
Val	1.6
Met	0.1
Ile	3.6
Leu	1.3
Tyr	4.2
Phe	1.4
Lys	0.5
His	0.1
Arg	1.2

^a^ Tryptophan could not be detected due to its hydrolytic structure was destroyed by hydrochloric acid. In the hydrolysis process, amino acids Asn and Gln were changed to Asp and Glu, respectively.

### 3.4 FT-IR spectrum analysis

Infrared spectroscopy is an essential tool for investigating polysaccharide structure, as specific functional groups such as -OH, C-O, C-O, and -NH_2_ in carbohydrate chains display distinctive absorption peaks in the range of 4,000–400 cm^−1^ (Guan et al., 2022). Figure 2C shows the FT-IR spectrum of LBNP-1, highlighting the characteristic carbohydrate peaks. Notably, a strong and broad absorption peak between 3,500 and 3,000 cm^−1^ corresponded to the O-H stretching vibration of hydroxyl groups, and the absorbance at 2,942 cm^−1^ was attributed to the C-H stretching vibration of hydrocarbon groups, representing characteristic absorption bands of polysaccharides. The band at approximately 1643 cm^−1^ indicated the presence of bound water (Wang Y.-X. et al., 2022). The absorbance band at 1,027 cm^−1^ indicated the bending vibrational mode of typical C-O-C stretching in pyranose (Li N. et al., 2022). Consistent with the aforementioned data, all these absorption bands confirmed the neutral polysaccharide moiety contained in LBNP-1.

### 3.5 Methylation analysis

To determine the type of linkage between glycosyl residues of LBNP-1, methylation analysis was carried out. The GC‒MS analysis results are presented as the linkage patterns of the monosaccharides in Table 3. The saccharide moiety of LBNP-1 primarily consisted of four glycosidic linkage patterns: t-Ara*f*, 1,2-Ara*f*, 1,2,5-Ara*f*, and t-Gal*p*. The most abundant linkage pattern was 1,2-Ara*f,* accounting for 59.05% of the composition, indicating the presence of a 1,2-arabinan backbone in LBNP-1. Additionally, a small portion of the 1,2-Ara*f* residue was branched at the *O*-5 position, most likely due to the presence of the t-Gal*p* residue, suggesting the branched nature of galactoarabinan. The presence of high amounts of terminally linked Ara*f* residues could be attributable to the peeling effect in the methylation reaction. In summary, LBNP-1 is a galatoarabinan contained glycoprotein.

**TABLE 3:** Methylation analysis of LBNP-1.

Deduced linkage	Peak	Molar ratio (%)	Mass fragments (m/z)	Type of linkage
1,4-di-O-acetyl-2,3,5-tri-O-methyl arabinitol	1	30.08	71, 87, 102, 118, 130, 161	t-Ara*f*
1,2,4-tri-O-acetyl-3,5-di-O-methyl arabinitol	2	42.04	71, 87, 101, 121, 161, 190	1,2-Ara*f*
1,2,4,5-tetra-O-acetyl-3-O-methyl arabinitol	3	17.01	71, 87, 101, 129, 155	1,2,5-Ara*f*
1,5-di-O-acetyl-2,3,4,6-tetra-O-methyl galactitol	5	10.87	71, 87, 102, 118, 145, 161, 205	t-Gal*p*

### 3.6 NMR analysis of LBNP-1

NMR spectra were employed to analyze the chemical shifts of characteristic features of polysaccharides present in the repeating units. Supplementary Figure S1 shows the ^1^H and ^13^C NMR spectra. The ^1^H NMR spectrum displays the typical chemical shift features of polysaccharides, which range from δ 3 to 5 ppm. Signals at around δ 4.0 and 4.1 ppm may be indicate the protons of the N-acetyl group of the protein bound residues. ^1^H NMR signals detected at 4.7 ppm were ascribed to the proton of water (H_2_O). Signals of ^1^H NMR at 3.7 and 3.8 ppm were assigned to H-2 and H-3 of 1,2-Ara*f* residue, respectively. Due to the relatively low total sugar content of LBNP-1 obtained, the ^13^C spectrum signal was weak, but two primary anomeric carbon signals could still be seen at δ 100 and 79 ppm, suggesting that LBNP-1 included two residues. Qin et al. (2021) isolated glycoprotein (PGP1, PGP2 and PGP3) from the pea (*Pisum sativum* L.) and identified its structure. Unfortunately, due to the lower total sugar content of PGP1, PGP2, and PGP3 compared to the protein content, the fine structure of glycoprotein was not presented as a result. Moreover, according to the literature, we found that most glycoprotein, such as those isolated from *Sipunculus nudus*, *Porphyra haitanensis* were also reported to have not obtained a fine structure (Lu et al., 2023; Ou et al., 2023). These results reminded us that the relatively low total sugar content of glycoprotein prevents the acquisition of the fine structure of polysaccharide proteins.

### 3.7 The effect of LBNP-1 on RAW264.7 cell viability

**FIGURE 3:**
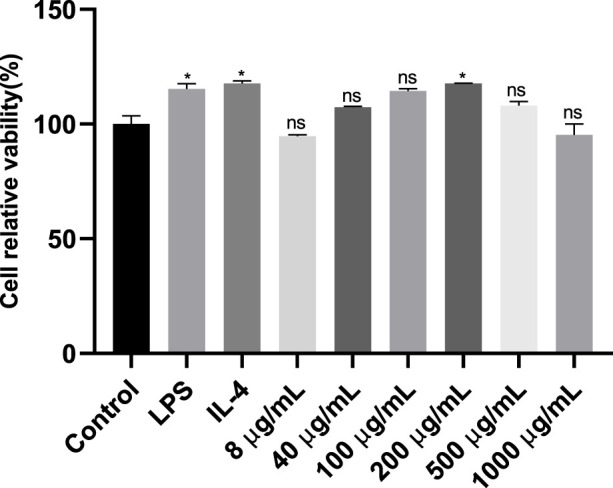
Analysis of the inhibitory effects of different doses (8, 40, 100, 200, 500 and 1,000 μg/mL) of LBNP-1 on macrophage proliferation after 24 h of treatment using a Cell Counting Kit-8 (CCK-8) assay. The data represent the mean ± SD of three independent experiments. *n* = 3, *P < 0.05, **P < 0.01, ns is not significant.

The toxicity of various doses of LBNP-1 on RAW 264.7 cells was evaluated using the CCK-8 method (Figure 3). Compared to the negative control, LBNP-1 did not significantly affect the proliferation of RAW264.7 cells within the concentration range of 8–1,000 μg/mL, indicating that LBNP-1 did not exhibit cytotoxicity toward macrophages. Therefore, we chose LBNP-1 (100 μg/mL) as the subsequent experimental dose.

### 3.8 LBNP-1 reverses the tumor-associated macrophage phenotype

**FIGURE 4:**
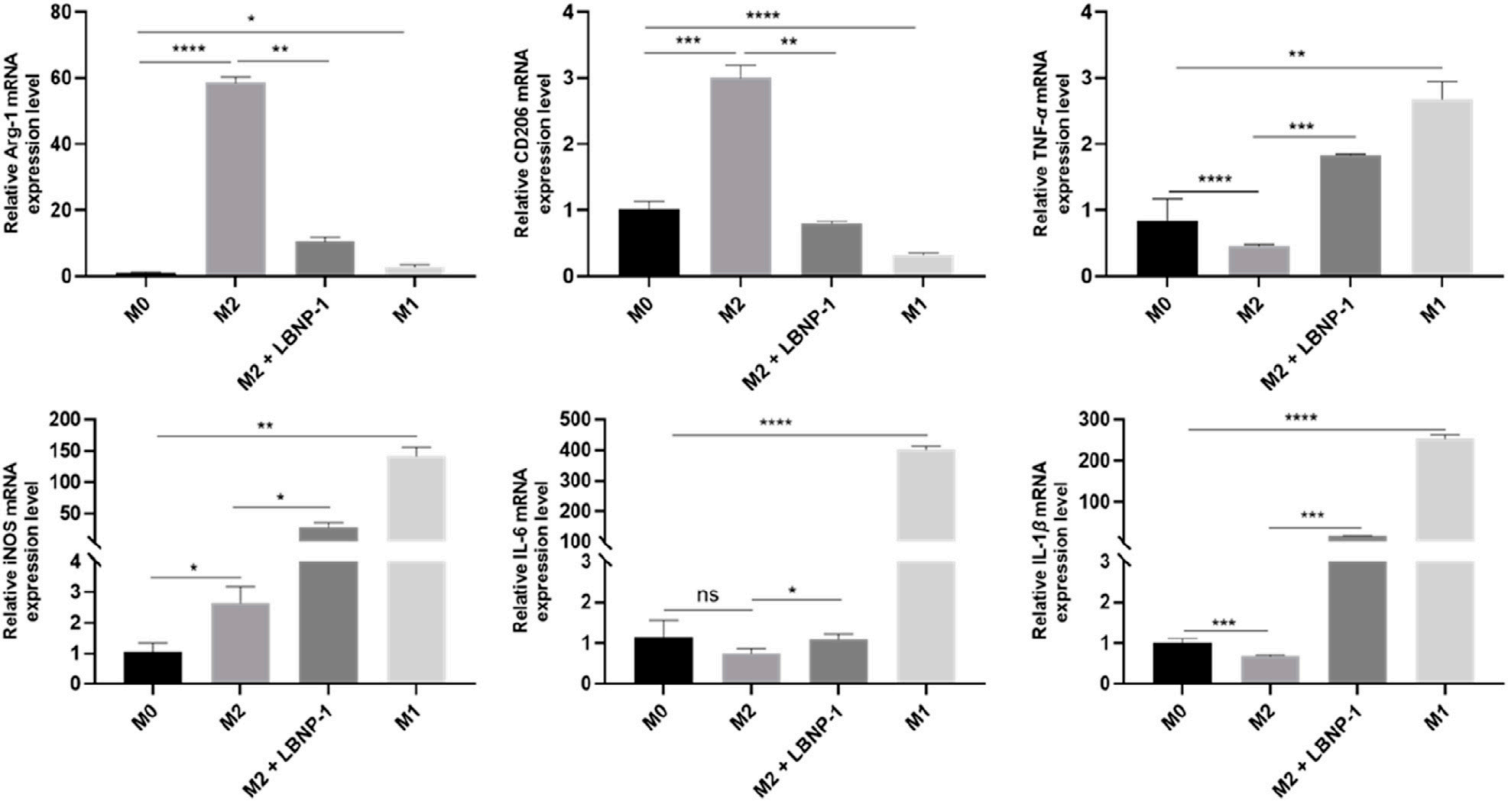
Effect of LBNP-1 on the induction of M2 macrophages to transform into the M1 phenotype *in vitro*. Error bars represent the S.D. (*n* = 3 independent experiments). *P < 0.05, **P < 0.01, ***P < 0.001, ****P < 0.0001; ns, not significant.

When RAW264.7 cells are treated with cytokines, they can be induced to differentiate into different types of macrophages. Macrophage polarization is accompanied by significant changes in gene expression. To preliminarily evaluate the antitumor effect of neutral polysaccharide, an *in vitro* polarization model of macrophages was established by optimizing the polarization time and cytokine concentration. The effect of LBNP-1 (100 μg/mL) on the expression of marker genes related to M1- and M2-like macrophages was analyzed. As depicted in Figure 4, stimulation with IL-4 noticeably increased the mRNA expression of Arg-1 and CD206, while the presence of LPS significantly elevated the expression of TNF-*α*, iNOS, IL-6, and IL-1*β*. These results confirmed the effective induction of both M1- and M2-phenotype macrophages. Following treatment with LBNP-1, the mRNA expression levels of Arg-1 and CD206 in M2 macrophages were significantly suppressed. Furthermore, the levels of IL-6, IL-1*β*, TNF-*α*, and iNOS were increased. These findings suggest that LBNP-1 can transform RAW264.7-polarized M2-like macrophages into M1-like macrophages.

### 3.9 Determination of PRRs

**FIGURE 5:**
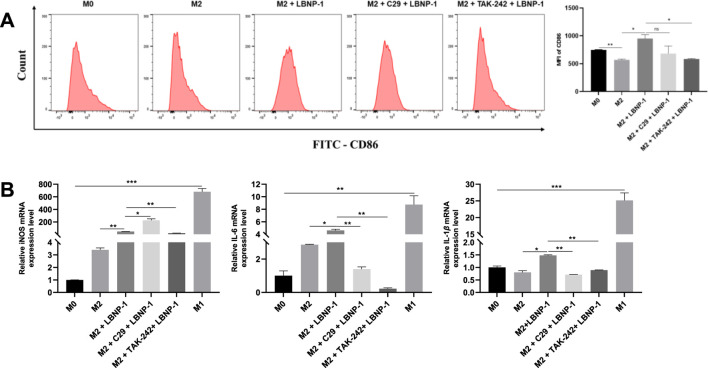
The significance of C29 and TAK-242 receptor blockers in LBNP-1 induction. **(A)** Expression of the M1 marker CD86 was detected by flow cytometry; **(B)** iNOS, IL-6, and IL-1*β* were analyzed by RT-PCR. Error bars represent the S.D. (*n* = 3 independent experiments). *P < 0.05, **P < 0.01, ***P < 0.001; ns, not significant.

TLRs, specifically TLR2 and TLR4, are common polysaccharide receptors. To determine whether TLR2 or TLR4 are involved in LBNP-1-induced macrophage activation, the receptor-specific blockers C29 and TAK-242 were utilized. M2-like RAW 264.7 cells were incubated with the TLR4 inhibitor TAK-242 (10 μM) or the TLR2 inhibitor C29 (10 μM) for 2 h prior to LBNP-1 treatment. As shown in Figure 5A, the expression of CD86 (an M1 marker) in M2 macrophages increased after LBNP-1 treatment, indicating that LBNP-1 could convert M2 macrophages into M1 macrophages. In addition, both a TLR4 inhibitor (TAK-242) and a TLR2 inhibitor (C29) significantly blocked this effect, suggesting the involvement of both TLR4 and TLR2 in the repolarization effect of LBNP-1 on M2 macrophages. Furthermore, treatment with TLR4 or TLR2 inhibitors notably decreased the expression of iNOS, IL-6, and IL-1*β* mRNA (Figure 5B), particularly in the TLR4 inhibitor group. These results indicate that TLR4 may be a key receptor for LBNP-1 on macrophages and may play an essential regulatory role in macrophage polarization. TLR2 also contributes to the repolarization effect, albeit to a lesser extent.

### 3.10 Effect of LBNP-1 on the NF-*κ*B signaling pathway

**FIGURE 6:**
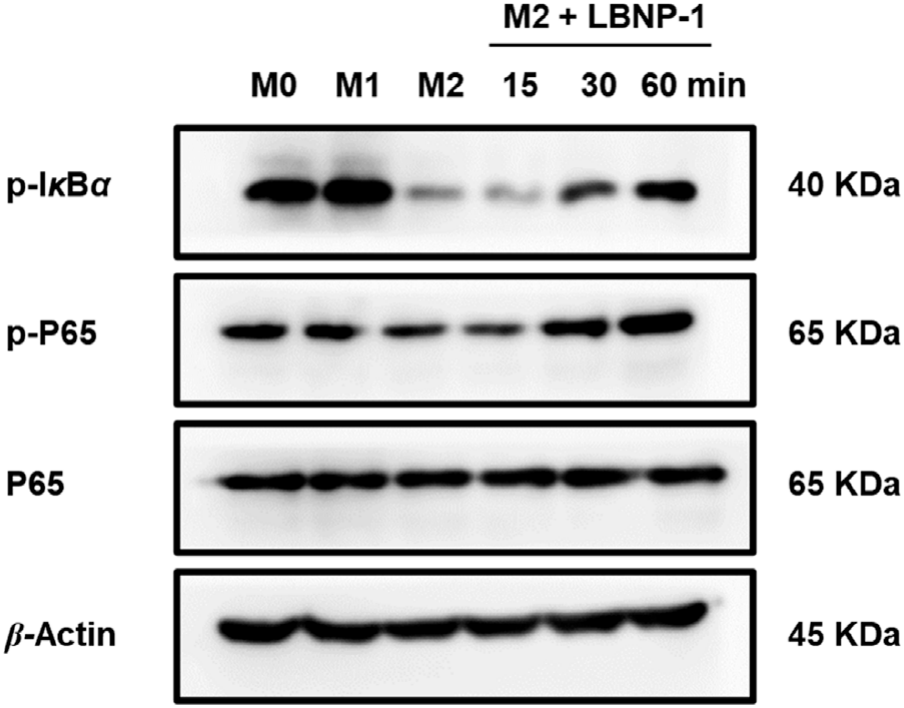
Effect of LBNP-1 on the protein expression of key nodes in the NF-*κ*B signaling pathway in M2 macrophages. M1 was used as a positive control.

To confirm whether NF-*κ*B is involved in LBNP-1-mediated macrophage polarization, the phosphorylation of I*κ*Bα and P65, which are vital members of the NF-*κ*B signaling pathway regulating transcriptional activity, was assessed. The results demonstrated a significant increase in p-I*κ*Bα and p-P65 levels in M2 macrophages treated with LBNP-1 (100 μg/mL) over time (Figure 6). These findings suggest that activation of the NF-*κ*B signaling pathway is at least partly responsible for the repolarization of M2 macrophages toward the M1 phenotype induced by LBNP-1.

### 3.11 Conditional medium from M2 macrophages treated with LBNP-1 inhibits MC-38 cell proliferation

**FIGURE 7:**
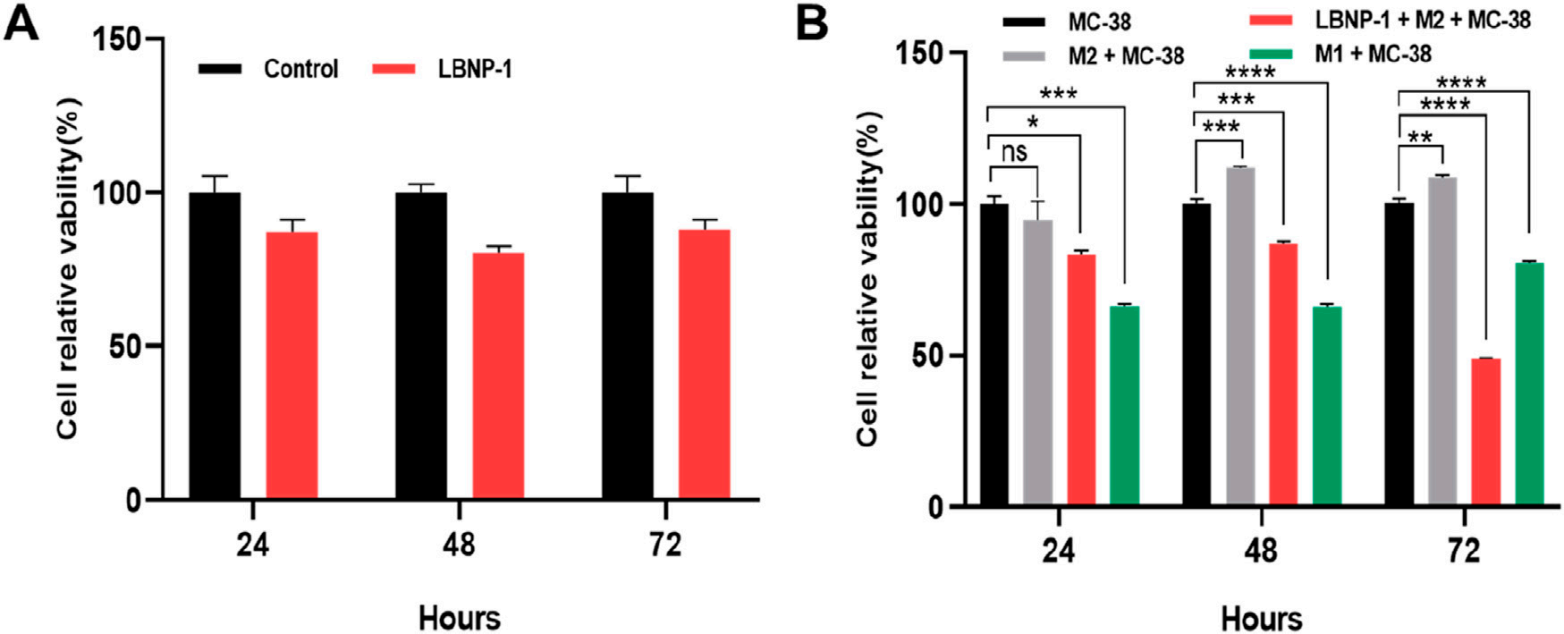
Activation of CM by LBNP-1 inhibits the proliferation of MC-38 cells. **(A)** The relative viability of MC-38 cells was measured by the CCK-8 method. **(B)** Effect of conditioned medium on the proliferation of MC-38 cells. Error bars represent the S.D. (*n* = 3 independent experiments). *P < 0.05, **P < 0.01, ***P < 0.001, ****P < 0.0001; ns, not significant.

Initially, the effect of LBNP-1 on the viability of cancer cells was investigated. As depicted in Figure 7A, LBNP-1 did not exhibit direct cytotoxicity toward MC-38 colon cancer cells. To determine whether LBNP-1 exerts antitumor effects by promoting the transformation of M2 macrophages into M1 macrophages, the effect of conditioned medium from LBNP-1-treated M2 macrophages on MC-38 cells was evaluated. The results revealed that CM from M2 macrophages without LBNP-1 treatment had a minimal effect on the viability of MC-38 colon cancer cells at 24 h but significantly promoted proliferation at 48 and 72 h. Conversely, CM from LBNP-1-treated M2 macrophages significantly inhibited MC-38 colon cancer cell proliferation at 24, 48, and 72 h (Figure 7B). These *in vitro* experimental findings suggest that LBNP-1 can inhibit the proliferation of MC-38 colon cancer cells by inducing the polarization of M2 macrophages to the M1 phenotype.

## 4 Discussion

In the present study, a galactoarabinan-containing glycoprotein named LBNP-1, was obtained from the seed of the medicinal plant *L. barbarum*. Structural characterization of LBNP-1 included molecular weight determination, monosaccharide composition analysis, methylation studies, FT-IR and NMR spectra. Additionally, LBNP-1 was identified as an arabinogalactan contained glycoprotein with an average molecular weight of 56.2 kDa. Monosaccharide analysis revealed arabinose and galactose as predominant components at a 4.3:1.0 molar ratio. Immune modulation and antitumor activities of polysaccharides depend on molecular weight, morphology, branching patterns, and solubility (Yu et al., 2018). The inherent structural complexity and heterogeneity of polysaccharides complicate consistent characterization and structure-activity relationship studies (Wang B. et al., 2022). We isolated a water-soluble homogeneous glycoprotein fraction (LBNP-1) from *L. barbarum*. Comprehensive structural analysis necessitates identifying monosaccharide constituents, glycosidic linkages, and branching characteristics. As reported by Peng and Tian (2001), *L. barbarum* arabinogalactan possesses a (1→4)-*β*-*D*-galactan backbone with arabinofuranosyl residues substituted at *O*-3. Redgwell et al. (2011) discovered an arabinogalactan with a (1→3)-*β*-*D*-galactan backbone predominantly substituted at *O*-3 by Ara*f* residues. Wang et al. (2014) confirmed the structure of an arabinogalactan with a (1→6)-galactan backbone substituted at *O*-3 by galactosyl or arabinosyl groups. LBPA, an arabinogalactan, was isolated from the fruits of *L. barbarium* (Ningqi No. 4) (Yuan et al., 2016) and was described as a highly branched polysaccharide with a backbone of (1→6)-linked-*β*-*D*-galactopyranosyl residues. Many of these residues were substituted at the *O*-3 position by galactosyl, rhamnosyl, glucosyl, or arabinosyl groups. In our study, LBNP-1 contained a 1,2-arabinan that branched at the *O*-5 position of 1,2-Ara*f*, most likely via *t*-Gal*p* residues. These structural features differ from previously reported arabinogalactans, potentially reflecting variations in plant material, isolation methods, structural attributes, or analytical methodologies. Bioactivity assessment demonstrated LBNP-1 promotes M2-to-M1 macrophage repolarization and exerts antitumor effects. What structural determinants underlie these distinct immunomodulatory effects? Synthetic pseudo-tetrasaccharides mimicking *Streptococcus pneumoniae* type 6A capsule polysaccharides exhibited murine immunogenicity (Sukhova et al., 2021). From *Boswellia carterii*, Bahramzadeh et al. (2019) purified BCF1 and BCF2 polysaccharides composed of neutral sugars (63.7%–83.3%) and uronic acids (6.2%–22.3%). Molecular weights ranged from 295.5 to 504.7 kDa. While both displayed immunomodulatory activity *in vitro*, BCF2 more potently enhanced macrophage NO and cytokine production, likely due to its C-3 branched (1→6)-*β*-galactopyranosyl structure. Immunomodulatory efficacy of polysaccharides depends critically on molecular weight, sugar composition, glycosidic bond types, and branching features (Chen et al., 2022). Arabinogalactan is established as the principal immunomodulatory component of *L. barbarum* (Gong et al., 2018). LBNP-1, exhibiting antitumor activity, contains arabinose and galactose and has an average molecular weight of 56.2 kDa. Immunostimulatory capacity of polysaccharides is molecular weight-dependent (Feng et al., 2020). Furthermore, bioactivity is influenced by branching complexity (Zhang et al., 2021). Controlled trifluoroacetic acid hydrolysis of arabinogalactan side chains reduces immunostimulatory activity (Xu et al., 2023). Additionally, LBNP-1 is a glycoconjugate containing polysaccharide and protein moieties. Lectins specifically recognize carbohydrates and frequently possess multiple binding sites per polypeptide chain. Regulatory interactions between ganglioside GM1 pentasaccharide and galectin-1 dimers on neuroblastoma cells modulate growth (Siebert et al., 2003). Their NMR and computational methods enabled GM1 glycan analysis without isotope labeling. These methods showed utility for inhibiting tumor lectin functions, with broader applications beyond glycoscience. Terminal mannose, N-acetylglucosamine, or fucose moieties on cell-surface glycans are recognized by mannose receptors. Lectins detect mannose-α(2,3)-mannose epitopes, mediating complement-independent phagocytosis and enhancing immune defense (Lin et al., 2022). Thus, LBNP-1’s amino acid profile was quantified (Table 2). Detected amino acids included Asp, Ser, Glu, Pro, Ile, and Tyr (among 17 analyzed), aligning with Zhang et al. (2014). Glycoconjugate antitumor efficacy is associated with protein amino acid profiles, particularly Asp, Glu, and Gly abundance (Tao et al., 2009; Zhao et al., 2022). Glycan-binding proteins commonly feature Arg, Ser, Tyr, and Trp residues proximal to bound sialic acids, essential for ligand recognition (Zhang et al., 2016). Such amino acids enhance immune function, indicating glycoconjugates exhibit specific pharmacologic properties.

TAMs are crucial components of the TME and represent the most abundant population of immune cells infiltrating tumors. Unfortunately, rather than inhibiting tumors, the TME actually contributes to cancer initiation and tumor growth promotion (Bissell and Hines, 2011). Macrophages are highly adaptable cells that can exhibit either M1 or M2 phenotypes in response to different environmental stimuli. M1 macrophages possess tumor-killing capabilities and are typically polarized by Th1 cytokines and microbial stimuli. On the other hand, M2 macrophages are considered to be tumor-promoting and display a strongly immunosuppressive phenotype that supports tumor cell proliferation (Shapouri-Moghaddam et al., 2018). Consequently, M2 macrophages are attractive targets for cancer immunotherapeutics. Thus far, it has been widely accepted that converting M2 macrophages into M1 antitumor macrophages through the discovery of effective drugs is a promising therapeutic strategy for malignancies. Polysaccharides derived from various natural resources have extensive biological effects, especially in terms of strengthening or activating the immune response, leading to immunomodulatory and antitumor effects (Cai et al., 2022). Therefore, exploring the use of plant polysaccharides as a potent antitumor treatment strategy is highly important. In line with the findings of the literature, it has been suggested that polysaccharides such as those from *Ganoderma lucidum*, *Pseudostellaria heterophylla*, *Moringa oleifera* leaf, and *Cordyceps militaris* can induce the polarization of M2 macrophages to an M1 phenotype, resulting in an antitumor effect (Bi et al., 2020; Li T. et al., 2022; Pu et al., 2022; Wang et al., 2023). Considering these results, it is pertinent to investigate whether *Lycium* polysaccharide can directly bind to TAMs and promote M1 polarization. Colorectal cancer is one of the most prevalent forms of cancer worldwide, and a previous study demonstrated that LBP treatment (at concentrations of 250 and 500 μg/mL) reduced the expression of the NLRP3 inflammasome and its associated protein PYCARD while also inhibiting the secretion of inflammatory cytokines (Lian et al., 2023). In the present study, our aim was to determine whether LBNP-1 could exhibit antitumor activity through the reversal of the phenotype of tumor-associated macrophages.

Consistent with the findings of previous studies, our findings revealed that treatment with LBNP-1 increased the expression of M1 macrophage markers (iNOS, TNF-*α*, IL-6, and IL-1*β*) while simultaneously decreasing the expression of M2 macrophage markers (Arg-1 and CD206). These findings indicate that LBNP-1 has the potential to convert M2 macrophages to the M1 phenotype.

As direct binding sites for natural bioactive polysaccharides and other bioactive components, receptors play crucial roles in the regulation of diseases mediated by bioactive components. Polysaccharides are composed of multiple monosaccharides linked by glycosidic bonds. They have a relatively large molecular weight and cannot penetrate cells directly. Instead, they need to enter cells through cell membrane receptors. Pattern recognition receptors are considered the primary means of direct binding with macrophages to recognize polysaccharides. Therefore, polysaccharides must bind to cell membrane receptors and enter cells through receptor mediation to exert an innate antitumor response (Cui et al., 2020).

Among all known TLRs, TLR2 and TLR4 are the most critical receptors used by macrophages to recognize polysaccharides. Activation of these receptors can induce a tumoricidal effect by activating immune cells. Several polysaccharides, including *Cantharellus cibarius* polysaccharide, *Dendrobium officinale* polysaccharide, and *Cordyceps mili-taris* polysaccharide, have been shown to polarize TAMs toward the M1 phenotype by binding to the target receptor TLR2, thereby inhibiting multiple tumor growth processes (Bi et al., 2020; Meng et al., 2019; Wang H. Y. et al., 2022).

Our experimental study revealed that LBNP-1 reduced the mRNA level of M2-like macrophages by recognizing TLR4, primarily through the recognition of LBNP-1 by TLR4. Similar results were also obtained for other botanical polysaccharides. For example, polysaccharides extracted from apples have been found to inhibit tumor growth by targeting TLR4 receptors on TAMs (Sun et al., 2020). The NF-*κ*B signaling pathway, a classical signaling pathway, participates in the polarization of M2-like macrophages. Guo et al. reported that *Lepidium meyenii* polysaccharide can suppress M2-like macrophage polarization through the NF-*κ*B signaling pathway (Guo et al., 2020). Consistent with these findings, we observed that NF-*κ*B participated in the phenotypic reversal of M2 to M1 induced by LBNP-1. The NF-*κ*B signaling pathway was found to be crucial for macrophage polarization.

Growing evidence has shown that M2-type macrophages provide anti-inflammatory effects, promote tumor growth, and create conditions for tumor immune evasion. On the other hand, M1 macrophages can eliminate targeted tumor cells. According to the current research, the growth of tumor cells is not directly inhibited by LBNP-1. However, LBNP-1-containing conditioned medium significantly inhibited the growth of CRC cells through immunoregulatory mechanisms. These results demonstrated that conditioned medium triggered by LBNP-1 exhibited significant anticancer efficacy against CRC cells. Although LBNP-1 has shown anti-tumor effects *in vitro*, further validation is needed to determine whether it also exerts the same physiological effects in animal experiments *in vivo*. Collectively, these findings highlight the potential application of LBNP-1 as an immunomodulator for the treatment of malignancies.

## 5 Conclusion

In this study, we investigated the macromolecular structure of a galactoarabinan-containing glycoprotein, LBNP-1, which was purified from Ningxia *Lycium barbarum* L. fruit. Due to its relatively low sugar content, nuclear magnetic resonance was unable to provide a clear signal. Therefore, we characterized the structure of LBNP-1 as a galactoarabinan-containing glycoprotein. Its saccharide moiety was found to be composed mainly of 1,2-arabinose as the backbone component, which is different from previously described polysaccharides. Moreover, the amino acid constituents of LBNP-1 were quantified. Aspartic acid (3.71 mol%), serine (7.43 mol%), glutamic acid (4.12 mol%), proline (6.72 mol%), isoleucine (3.54 mol%), and tyrosine (4.23 mol%) constituted the predominant amino acid components of LBNP-1.

Our *in vitro* experiments demonstrated that LBNP-1 promoted the polarization of M2 macrophages to the M1 phenotype primarily through TLR4 receptors, thereby activating NF-*κ*B signaling pathways and alleviating the immunosuppressive microenvironment. Although LBNP-1 did not significantly suppress the growth of CRC cells *in vitro*, we observed that LBNP-1-containing CM inhibited CRC cell growth by inducing M2 macrophages to adopt the M1 phenotype. Future work should aim to further evaluate the antitumor effect of LBNP-1 *in vivo*.

## Data Availability

The datasets presented in this study can be found in online repositories. The names of the repository/repositories and accession number(s) can be found in the article/Supplementary Material.
