# Supplementary material for: A galactoarabinan-containing glycoprotein isolated from the fruits of Lycium barbarum reverses the tumor-associated macrophage phenotype
Source: Front Pharmacol. 2025 Oct 29;16:1593407. doi: 10.3389/fphar.2025.1593407 (PMC12605246; doi:10.3389/fphar.2025.1593407)
Supplement: Supplementary file 1 [file Supplementaryfile1.docx]

**Supplementary Data**

|  |
| --- |
|  |

Fig. S. ^1^H (A)and ^13^C(B) NMR spectra of LBNP-1.
